# Prognostic Value of Ki67 Percentage, WT-1 Expression and p16/CDKN2A Deletion in Diffuse Malignant Peritoneal Mesothelioma: A Single-Centre Cohort Study

**DOI:** 10.3390/diagnostics10060386

**Published:** 2020-06-09

**Authors:** Federica Pezzuto, Gabriella Serio, Francesco Fortarezza, Anna Scattone, Concetta Caporusso, Alessandra Punzi, Domenica Cavone, Antonio Pennella, Andrea Marzullo, Luigi Vimercati

**Affiliations:** 1 Department of Cardiac, Thoracic, Vascular Sciences and Public Health, University of Padova, 35128 Padova, Italy; francescofortarezza.md@gmail.com; 2 Pathology Division, Department of Emergency and Organ Transplantation, University of Bari, 70124 Bari, Italy; kcaporusso.c@libero.it (C.C.); alessandra.punzi@hotmail.it (A.P.); andrea.marzullo@uniba.it (A.M.); 3 Pathology Division, IRCCS National Cancer Institute “Giovanni Paolo II”, 70124 Bari, Italy; a.scattoneanatopat@alice.it; 4 Occupational Health Division, Department of Interdisciplinary Medicine, University of Bari, 70124 Bari, Italy; domenica.cavone@uniba.it (D.C.); luigi.vimercati@uniba.it (L.V.); 5 Pathology Division, Department of Surgery, University of Foggia, 71122 Foggia, Italy; antonio.pennella@unifg.it

**Keywords:** diffuse malignant peritoneal mesothelioma, prognostic factors, Ki67, WT-1, p16, CDKN2A

## Abstract

Diffuse malignant peritoneal mesothelioma (DMPM) is a rare malignant neoplasm with a poor survival. Although some advances in knowledge have been obtained for the pleural form, much less is known about DMPM. Advantages in terms of prognosis are still limited and strong efforts need to be made. The aim of our study was to correlate several histological and molecular factors with survival in a large cohort of 45 DMPMs. We evaluated histotype, nuclear grade, mitotic count, necrosis, inflammation, desmoplastic reaction, Ki67 percentage, WT-1 expression, p16 protein by immunohistochemistry and CDKN2A deletion by FISH. Our results showed that epithelioid histotype, nuclear grade 2, mitotic count ≤5 x mm^2^, absence of desmoplasia and p16/CDKN2A deletion, low Ki67 value, and high WT-1 expression were correlated with the most prolonged survival (*p* = 0.0001). Moreover, p16 loss in immunohistochemistry reflected CDKN2A deletion detected with FISH, and both were correlated with the worst survival (*p* = 0.0001). At multivariate analysis, Ki67 value, WT-1 expression and p16/CDKN2A deletion emerged as independent prognostic factors (*p* = 0.01, *p* = 0.0001 and *p* = 0.01, respectively). These parameters are easy to analyse at the time of DMPM diagnosis and may support better patient stratification, prediction of treatment effectiveness and therapeutic optimization.

## 1. Introduction

Malignant mesothelioma (MM) is a rare neoplasm that arises from mesothelial cells of serous cavities [1]. The onset of MM is strictly related to asbestos exposure, and there is a long latency time prior to first manifestations. Thus, based on the widespread employment of asbestos in the past years, the incidence is expected to drastically increase, reaching a peak between 2002–2050 [2]. MM remains a very aggressive and fatal disease. If untreated, the five-year mortality rate is higher than 95% [3]. Currently, therapeutic strategies are based on a multimodal approach, including surgery, radiation, and chemotherapy. More recently, targeted therapy with biological agents (i.e., tyrosine kinase inhibitors, proteasome inhibitors) [4] and immunotherapy with immune checkpoint inhibitors (i.e., anti -programmed cell death 1-PD-1/anti -programmed cell death ligand 1 PD-L1 drugs) [5] have also been attempted, but the results are disappointing. Multiple factors have been shown to be significant in predicting outcome and overall survival. In this regard, a new proposal by the European network for Rare adult solid Cancer (EURACAN)/ International Association for the Study of Lung Cancer (IASLC) has also been designed in order to update the histologic classification of MM, from the perspective of a multidisciplinary approach [6]. Most studies have been conducted and advances obtained for the pleural form while much less is known about the diffuse malignant peritoneal mesothelioma (DMPM) that accounts for 5–10% of all MMs [7]. Despite many histological similarities, peritoneal and pleural tumours show quite different clinical and prognostic features. In this regard, a reliable staging system has not yet become available. Indeed, unlike the pleural counterpart, DMPM still lacks a standardised method for defining the prognosis of the disease based on its extension. A tumour-node-metastasis (TNM) staging system for DMPM was proposed in 2011, that yielded a significant stratification of survival [8]. However, the rarity of the disease, the ineffectiveness of therapies and the natural history of this tumour did not promote wide acceptance of this system.

DMPM shows a highly variable biological aggressiveness. The morbidity and mortality of the disease are mainly related to progressive locoregional effects in the abdomen. Standard treatment consists of cytoreductive surgery and hyper-thermic intraperitoneal chemotherapy in resectable disease, and standard chemotherapy regimens in inoperable stages. As the advantages in terms of prognosis are still limited, strong efforts should be made to achieve better patient stratification, improving the prediction of treatment effectiveness and optimising therapies. The identification of standard prognostic factors may bring us a step forward in supporting therapeutically relevant conclusions. To date, few studies have been focused on DMPM and the results are incomplete [9,10,11]. The aim of our study was to correlate several histological (histotype, nuclear grade, mitotic count, necrosis, inflammation, desmoplastic stromal reaction, proliferative index, Wilms’ Tumour 1-WT-1 and p16 expression) and molecular factors (cyclin-dependent kinase inhibitor 2A-CDKN2A deletion) with survival in a large cohort of 45 DMPMs in order to determine their possible prognostic and predictive role of these factors.

## 2. Materials and Methods

The present study was carried out on 45 cases of peritoneal mesothelioma samples consecutively diagnosed at the Pathology section of the University of Bari between March 1990 and May 2012. Patients’ epidemiological data on asbestos exposure (occupational and/or environmental), clinical data (sex, age, stage of the disease), date of diagnosis, and survival were collected through questionnaires or family interviews and classified according to the criteria of the National Mesothelioma Register criteria, and encoded in an electronic database. The study was approved by the local Ethics Committee of the Policlinico-Hospital, Bari, Italy (accession number 5062, 22 June 2016).

Histological examination was performed of 43 multiple laparoscopic biopsies, an autoptic sample, and a surgical intestinal resection. In four patients the onset of the disease was in a hernial sac. All patients were chemo-naïve, and none had received cytoreductive surgical therapy and/or intraperitoneal chemotherapy. After diagnosis, three patients had received no treatment, one patient underwent surgery, and in most patients only palliative platinum-based chemotherapy was administered. All biopsy specimens were fixed in 10% buffered neutral formalin and paraffin embedded. Histological sections were stained with haematoxylin-eosin and assessed for: (a) histotype (epithelioid, biphasic and sarcomatoid), (b) nuclear grade (1, 2, 3 following Kadota et al. [12]), (c) mitotic count (expressed on mm^2^), (d) necrosis, inflammatory infiltrate, and desmoplasia (absent or present). For immunohistochemistry, a wide panel of antibodies was used for diagnosis, following WHO recommendations [1]. Ki67 was evaluated by MIB-1 (clone K5001, DAKO, dilution 1:200) antibody. For WT-1 detection a mouse monoclonal antibody (clone WT49, Novocastra, dilution 1:40) was used. Ki67 and WT-1 were expressed as the percentage of positive cells in total cell number. Positivity was also classified as low (≤25% and ≤15%, respectively) and high (>25% and >15%, respectively) based on the median value.

For p16 detection a rat monoclonal antibody (clone G175-405, BD Pharmingen, dilution 1:50) was used. The p16 expression was assessed in both the number of positive cells and the intensity of cytoplasmic and/or nuclear staining (score 0: absent, score 1: focal or diffuse low intensity staining, score 2: diffuse and moderate intensity staining, score 3: diffuse and high intensity staining). P16 was considered positive only for scores ≥2 [13].

A FISH locus specific CDKN2A (9p21) probe (Abbott, Abbott Park, IL, USA) was used to detect chromosome 9 deletion. Vysis LSI CDKN2A/CEP 9 probes are provided in one vial as a mixture of the LSI CDKN2A (p16) probe labelled with Spectrum Orange and the CEP 9 probe labelled with Spectrum Green. The LSI CDKN2A probe spans approximately 222 kb and contains several genetic loci including D9S1749, DS1747, p16 (INK4B), p14 (ARF), D9S1748, p15(INK4B), and D9S1752. The CEP 9 Spectrum Green probe hybridizes to alpha satellite sequences specific to chromosome 9, CE Marked. At least 100 cells were scored for each case. A cut-off of 20% was used for homozygous deletion. Heterozygous deletion was defined as when >20% of the cells showed only one signal or lower signal number than CEP9.

A multivariate analysis was performed, including only those variables that resulted statistically significant at univariate analysis (histotype, nuclear grade, mitotic count, desmoplastic stromal reaction, Ki67 value, WT-1 percentage, p16/CDKN2A deletion).

Survival was calculated considering the interval between the day of diagnosis and death or the last follow-up. For statistical analyses, SPSS software, Inc.; Chicago, IL, version 11 was used. Survival curves were calculated with the Kaplan-Meyer method and significance with the Log-Rank test. For the entire data set, multivariate analysis was performed using Cox regression analysis. Pearson’s analysis was used for statistical correlations. *p* values less than 0.05 in the two-tailed analyses were considered to denote statistical significance.

## 3. Results

Forty-five DMPMs were evaluated. 35 male (77.8%) and 10 female (22.2%) patients with a mean age of 63.7 ± 11.5 years were included in the study. Asbestos exposure was known in 28 of the 45 patients (62.2%). Clinical data were collected for all patients. 

### 3.1. Histologic and Molecular Analysis

#### 3.1.1. Histologic Evaluation

Tumour histotypes included 32 (71.1%) epithelioid, 9 (20%) biphasic and 4 (8.9%) sarcomatoid mesotheliomas. Nuclear grade 1 was never detected, while nuclear grade 3 (32 cases, 71.1%) was the most frequent. Mitotic count showed a mean of 7.6 ± 5.6 x mm^2^. Necrosis, inflammatory infiltrate and desmoplastic reaction were present in 18 cases (40%), 34 cases (75.6%) and 15 cases (33.3%), respectively, showing a high variability in distribution. The mean of Ki67 percentage was 24.6 ± 15.8% for epithelioid, 36.9 ± 17% for biphasic and 49.8 ± 10.5% sarcomatoid histotypes. A WT-1 >15% was detected in 19 (42.2%) patients(Table 1). 

#### 3.1.2. P16 Expression and CDKN2A Status

Immunohistochemical evaluation for p16 showed scores 2–3 in 22 of the 45 cases (48.9%) and score 0–1 in 23 cases (51.1%). Positivity for p16 (score 2–3) was observed in 18 epithelioid histotypes, in 4 biphasic and in only 1 sarcomatoid mesothelioma. FISH analysis for p16/CDKN2A revealed absence of deletion in 8 cases (17.8%), heterozygous deletion in 14 cases (31.1%) and complete homozygous deletion in 23 cases (51.1%). The CDKN2A deletion was more frequently detected in females (*p* = 0.005), biphasic/sarcomatoid histotype (*p* = 0.007), high nuclear grade (*p* = 0.05), high mitotic count (*p* = 0.006), high proliferative index (*p* = 0.015) and low WT-1 expression (*p* = 0.014). No correlation was found between CDKN2A deletion and asbestos exposure. Interestingly, all 23 patients with negative p16 staining also showed a homozygous deletion by FISH analysis (Table 1).

#### 3.1.3. Analysis of Survival

Mean survival was 15.3 ± 2.6 months. To assess whether there was a time bias in terms of survival related to the time of recruitment, we considered two periods (from 1990 to 2003 and from 2004 to 2012). In fact, in 2003 a crucial step towards new therapy in malignant mesothelioma was made. Vogelzang et al. published the results of a phase III randomized trial, showing a higher rate of objective clinical response for the drug combination pemetrexed-cisplatin (41% vs. 17%) when compared to the cisplatin-only group [14]. Although this study was carried out on pleural mesothelioma, because the effectiveness of chemotherapeutic agents could be considered similar also for peritoneal forms [15], this study revolutionised mesothelioma treatment, bringing about a new standard of chemotherapeutic care. In any case, comparison between the groups before and after this data showed comparable mean survival for the two groups (12.2 vs. 12.4 months, respectively).

**Figure 1:**
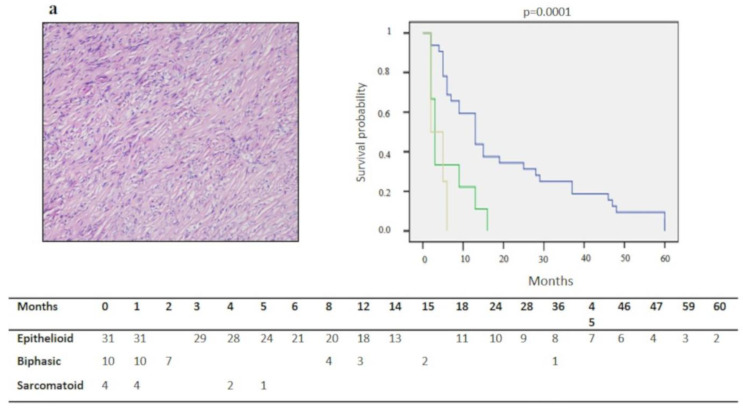

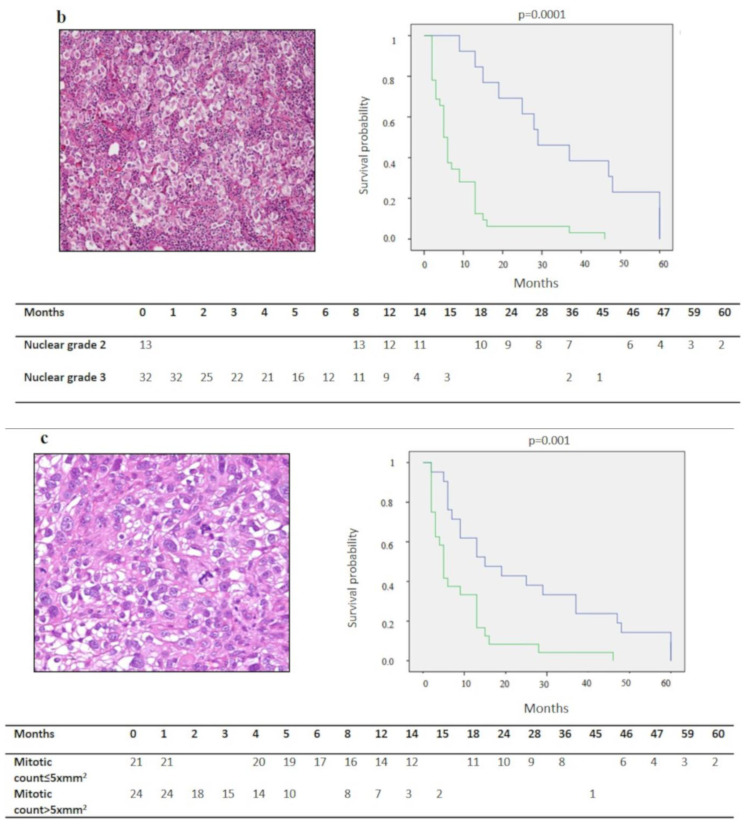

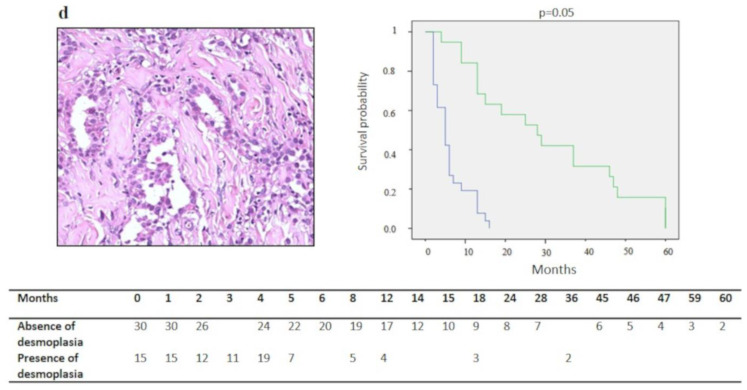
Correlation between histological variables and survival. Sarcomatoid histotype (hematoxylin and eosin stain, original magnification ×200). (**a**), nuclear grade 3 (hematoxylin and eosin stain, original magnification ×200); (**b**), mitotic count >5 x mm^2^ (hematoxylin and eosin stain, original magnification ×200); (**c**) and prominent desmoplastic stromal reaction (hematoxylin and eosin stain, original magnification ×400); (**d**) showed a shorter survival when compared with their counterparts (Green lines for: biphasic histotype in (**a**), nuclear grade 3 in (**b**), mitotic count >5 x mm^2^ in (**c**), absence of desmoplasia in (**d**). Blue lines for: epithelioid histotype in (**a**), nuclear grade 2 in (**b**), mitotic count ≤5 x mm^2^ in (**c**), presence of desmoplasia in (**d**). Yellow line for sarcomatoid histotype in (**a**)).

**Figure 2:**
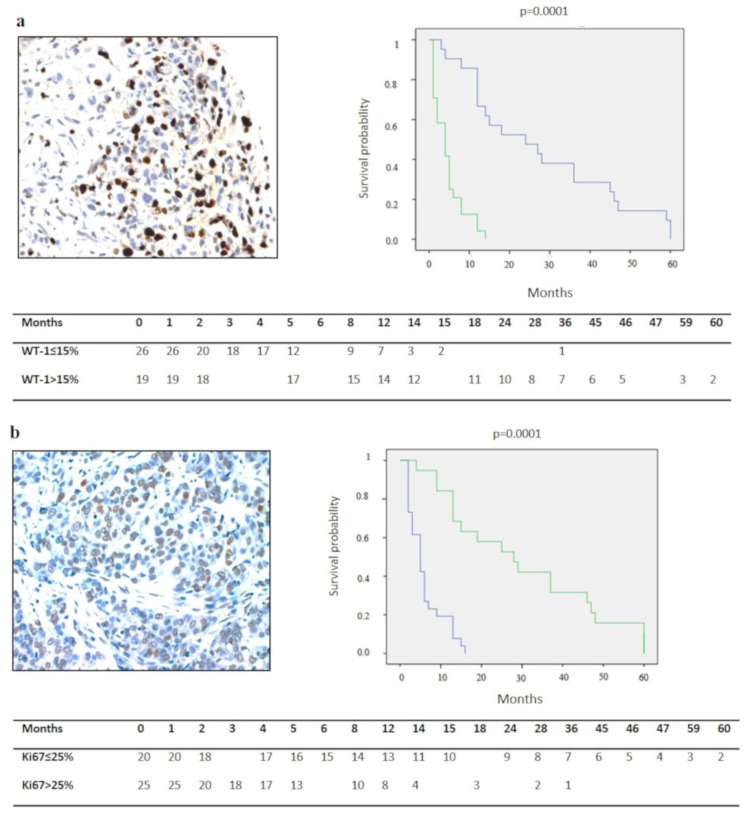

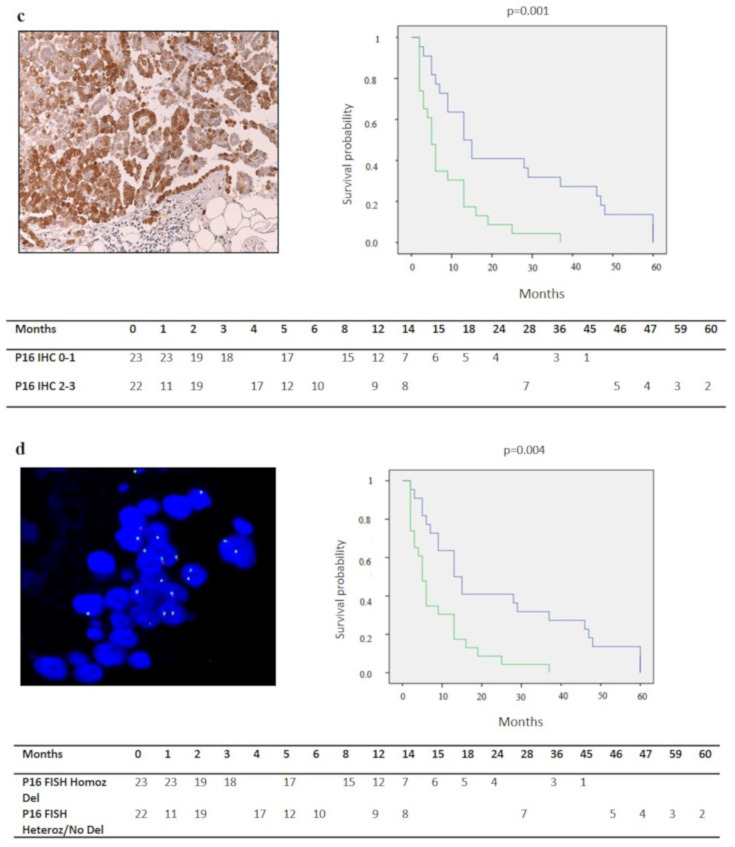
Correlation between immunohistochemical and molecular variables and survival. Ki67 > 25% (immunohistochemistry, original magnification ×400). (**a**) and WT-1 ≤ 15% (immunohistochemistry, original magnification ×400); (**b**) showed a shorter survival, whereas high p16 immunostaining score (immunohistochemistry, original magnification ×100); (**c**) and/or absence or heterozygous CDKN2A deletion showed a longer survival when compared with their counterparts; (**d**) (Green lines for: Ki67 > 25% in (**a**), WT-1 > 25% in (**b**), score 0–1 of p16 in (**c**), homozygous deletion of CDKN2A in (**d**). Blue lines for: Ki67 ≤25% (**a**), WT-1 ≤25% in (**b**), score 2–3 of p16 in (**c**), no/heterozygous deletion of CDKN2A in (**d**)).

A statistically significant difference was detected for survival in epithelioid, biphasic and sarcomatoid DMPMs (19.7 ± 3.3 vs. 4.8 ± 1.8 vs. 2.7 ± 1 month, *p* = 0.0001) (Figure 1a). Cases with nuclear grade 2 (Figure 1b), mitotic count ≤5 x mm^2^ (Figure 1c), no desmoplasia (Figure 1d) (*p* = 0.0001), Ki67 ≤ 25% (*p* = 0.0001) (Figure 2a) WT-1 >15% (*p* = 0.0001) (Figure 2b), p16 immunohistochemical expression scores 2–3 (*p* = 0.0001) (Figure 2c) and no deletion or heterozygous deletion of CDKN2A (*p* = 0.0001) (Figure 2d) showed a better survival than their counterparts (*p* = 0.0001). At the multivariate analysis, Ki67 percentage, WT-1 expression and p16/CDKN2A deletion emerged as independent predictors of survival (*p* = 0.01, *p* = 0.001, *p* = 0.017, respectively) (Table 1).

## 4. Discussion

DMPM is a tumour with a poor prognosis. To date, conventional treatment strategies (chemotherapy, surgery, intraperitoneal chemotherapy) provide only limited benefits for prolonging survival. However, rare cases of patients with long-term survival have been reported in the literature [16], thus supporting the biological variability of this tumour.

The evaluation of prognostic parameters showing a correlation with prognosis is of primary importance in the management of patients with DMPM. In the present study several pathological and biological parameters were analysed and correlated with survival in order to predict the clinical outcome. Evaluation of the histological features confirmed that the epithelioid histotype was the most frequent, and correlated with the longest survival, in accordance with the literature reports. Indeed, patients with sarcomatoid and biphasic types have a higher risk of mortality [17].

Mitotic count and nuclear grade have been proposed as reliable prognostic factors in patients with mesothelioma [9,10]. In our study, all samples showed nuclear grades 2 and 3, and cases with nuclear grade 3 were correlated with a significantly lower survival. Similarly, a high mitotic count was associated with a poor prognosis and a survival for less than one year. Furthermore, from the results of our research, desmoplastic reaction was associated with a poor prognosis. Interestingly, this finding seems to be in line with the recent proposals in pleural MM [6].

As in other malignancies, DMPM Ki67 has also been demonstrated to be an important prognostic marker, alone or combined to other factors [18,19]. It has also been suggested to predict survival in patients treated with combined therapies [20]. Our results confirmed these previous findings.

WT-1 expression is conventionally used as a positive diagnostic marker in MM [21]. The first study on the prognostic significance of WT-1 in mesotheliomas carried out by Kumar-Singh et al. [22] did not show a correlation with prognosis. Scattone et al. suggested a positive prognostic significance of WT-1 expression in epithelioid peritoneal mesotheliomas, with a low mitotic index and low nuclear grade [10]. Cedrés et al. demonstrated a significantly increased survival for patients with WT-1 positive expression [23]. Our results are in accordance with these findings, also when applying a two-tailed WT-1 value.

The CDKN2A alteration is quite frequent in MM [24]. The loss of p16 protein may be due to homozygous deletion, promoter methylation or point mutations. The highest frequency of mutations was associated with biphasic and sarcomatoid histotypes. Both homozygous and heterozygous deletion of p16 can be investigated by FISH analysis. P16 loss was studied for its diagnostic value in distinguishing between benign mesothelial and neoplastic proliferations. Illei et al. showed that CDKN2A deletion in FISH was a valid preliminary test on neoplastic cells from serous effusions, as the greatest number of deletions was observed in the malignant cells [25]. Chiosea et al. also confirmed the diagnostic utility of the marker on formalin-fixed and paraffin-embedded samples [26]. The prognostic implications of p16 loss was suggested by Borczuk et al. [8]. The authors showed that a biphasic histology, increased mitotic rate, and p16 loss were independently associated with poorer survival in DMPM. Some years later, a combination of preliminary p16 immunostaining with CDKN2A deletion identified patients with an unfavourable outcome after treatments [27]. Furthermore, homozygous CDKN2A deletions combined with hemizygous NF2 loss were found to be negative independent prognostic factors for both progression-free survival and overall survival in DMPM [10]. Our results are consistent with those reported in the literature, as patients with homozygous deletion showed the shortest survival.

In our research study, the homozygous deletion of p16/CDKN2A was detected in 23 of the 45 cases (51.1%). We have compared our results with those obtained from the genomic and molecular characterization of large series of pleural mesothelioma. Bueno et al. [28] reported chromosome rearrangement of tumour suppressor genes including CDKN2A in 45% of cases. This value was similar to the percentage reported by Hmeljak et al. (49%) [29]. The authors also found that loss of CDKN2A was strongly associated with a shorter overall survival. Interestingly, our findings are in line with these results, as regards both the incidence and the prognostic significance of the alteration.

In our study, CDKN2A deletion was not correlated with asbestos exposure. This association has been investigated in previous studies, but the results are controversial. Some works reported that deletion and asbestos exposure were not associated [30,31,32]. Instead, more recently, some authors suggested a different biology of the tumour in patients with or without asbestos exposure [24,33]. As no conclusive results have been obtained, this topic remains an important point warranting in-depth investigation.

In our research, one of the most striking observations is the strong correlation of immunohistochemical detection of p16 protein with CDKN2A deletion by FISH. Immunohistochemistry for p16 could be used as a first diagnostic step at the time of the diagnosis. However, a certain variability in immunohistochemical staining for all cases with a heterozygous deletion must be underlined. Thus, such cases should be scheduled for FISH to establish the homozygous deletion percentage.

In recent years, knowledge about the pathobiology of MM has dramatically increased, prompting investigations of several new treatments for both early stage and advanced stage disease setting. The heterogeneity of the neoplasia, as emphasised by large-scale molecular profiling studies [28,29], has limited the identification of specific biomarkers. Nevertheless, some molecules have been proposed as targets for personalized therapy, mostly for the pleural variant. In CDKN2A-mutated malignant pleural mesothelioma cell lines and in a mouse model, the inhibition of CDK4/CDK6 was shown to suppress cell viability and tumour growth [34]. This gave rise to a multi-arm stratified therapy based clinical trial for patients with CDKN2A negative relapsed mesothelioma. New treatment opportunities have also been defined for patients with WT-1 expressing malignant pleural mesothelioma. A WT-1 cancer vaccine made out of molecules similar to those in the WT-1 protein in combination with checkpoint inhibition is being tested in a phase I study [34]. In this context, our findings may contribute to clinical implications not only on prognosis but also on therapeutic strategies in DMPM.

This work included a large cohort of cases and allowed us to enrol a uniform population without any pre-treatment. Our study has some limitations. This was a single-centre retrospective study and specimens were collected over a long time period. Some important factors could not be evaluated during the study period, including data on asbestos exposure as well as specific chemotherapy schemes.

## 5. Conclusions

The present study was aimed at exploring the role of clinical-morphological and biological DMPM parameters that could be used to predict prognosis in clinical practice.

Our data confirmed the prognostic importance of the neoplastic histotype, nuclear grade, mitotic count and desmoplasia in affecting prognosis. Ki67 value, WT-1 and p16 immunohistochemistry are easily assessed at the time of diagnosis in DMPMs. These findings, matched with FISH analysis, could be useful in identifying those patients at highest risk for short survival and make a better prognostic stratification of patients with DMPMs. These tests could also be easily performed to select candidates for multimodal/aggressive therapeutic approaches or for inclusion in new clinical trials.

## Figures and Tables

**Table 1:** Results of statistical analyses of histological and molecular parameters.

Parameter	Number of Cases	Survival (Months) Mean ± SD (C.I. 95%)	*p*-Value	Multivariate Analysis
**Histotype**				
Epithelioid	32	19.7 ± 3.3 (13.2–26.2)		
Biphasic	9	4.8 ± 1.8 (1.3–8.4)	0.0001 *	*n.s*
Sarcomatoid	4	2.7 ± 1 (0.7–4.7)		
**Nuclear Grade**				
1	0			
2	13	33.7 ± 5.2 (23.5–44)	0.0001 *	*n.s*
3	32	76 ± 5.6 (4.3–11.1)		
**Mitotic Count**				
≤5/10 x mm^2^	21	23.5 ± 20.5 (14.7–32.2)	0.001 *	*n.s*
>5/10 x mm^2^	24	8 ± 10.1 (3.9–12.1)		
**Necrosis**				
Absent	27	17.3 ± 18.7 (10.3–24.4)	*n.s*	
Present	18	12.1 ± 15.4 (4.9–19.2)		
**Inflammatory Infiltrate**				
Absent	11	15.5 ± 19.1 (4.2–26.8)	*n.s*	
Present	34	15.1 ± 17.2 (9.3–21)		
**Desmoplastic Stromal Reaction**				
Absent	30	18.2 ± 19.2 (11.3–25.1)	0.05 *	*n.s.*
Present	15	9.3 ± 11.7 (3.3–15.2)		
**Ki67**				
≤25%	21	26.14 ± 3.5 (2.7–33.3)	0.0001 *	0.01 *
>25%	24	9.2 ± 1.5 (3.4–6.5)		
**WT-1**				
≤15%	26	5 ± 0.8 (3.4–6.7)	0.0001 *	0.001 *
>15%	19	29.2 ± 4.3 (20.6–37.8)		
**P16 IHC**				
Absent/≤5%	23	7.7 ± 1.8 (4.1–11.3)	0.001 *	0.017 *
Present	22	23.1 ± 4.4 (14.5–31.8)		
**P16 FISH**				
No deletion	8	22 ± 8.8 (4.6–39.3)		
Heterozygous deletion	14	23.8 ± 5.5 (13.9–33.8)	0.004 *	
Homozygous deletion	23	7.7 ± 1.8 (4.1–11.2)		

C.I.: confidence interval; *: statistical significance for *p* values less than 0.05; *n.s.*: not significant.
